# Is man the only animal with ears that cannot move them? Reflections on observational studies in obstetrics and gynecology

**DOI:** 10.1111/aogs.14925

**Published:** 2024-07-23

**Authors:** Lars Henning Pedersen, Zeyan Liew

**Affiliations:** ^1^ Department of Obstetrics and Gynecology Aarhus University Hospital Aarhus Denmark; ^2^ Department of Clinical Medicine Aarhus University Aarhus Denmark; ^3^ Department of Biomedicine Aarhus University Aarhus Denmark; ^4^ Department of Environmental Health Sciences and Yale Center for Perinatal, Pediatric, and Environmental Epidemiology, Yale School of Public Health New Haven USA

Aristotle noted almost 2500 years ago that “Man is the only animal with ears that cannot move them” (p. 14 of^1^). This statement, true or not, illustrates a profound emphasis on observation that is now the core of natural science: Aristotle based the claim on observations, not on a theoretical or theological system—even though it would be interesting to envision a belief that would come up with such a declaration.

In clinical medicine, however, pure observational studies are outranked by randomized controlled trials (RCTs). In the Oxford center for Evidence‐Based Medicine's (CEBM) Levels of Evidence, randomized controlled studies are level 1, and observational studies are level 2, 3, or 4, with only expert opinion ranking lower. Potentially bad news for older doctors and epidemiology—but the CEBM ranking comes with a brilliant preamble:

“What are we to do when the irresistible force of the need to offer clinical advice meets with the immovable object of flawed evidence? All we can do is our best: give the advice, but alert the advisees to the flaws in the evidence on which it is based.”

The core reason for the stronger trust in RCTs is causality. When we use medication, perform surgery, or conduct other interventions on humans, we want to make sure that it works. Trials give the opportunity to observe what would happen given an intervention if all else were equal. Epidemiology, the breeding ground of observational studies, offers plenty of examples of conclusion that have later proven to be wrong (however, far from as awful as has been put forward, see for example,^2^). “Association is not causation” while should have been made very clear in all modern epidemiology books, taught in entry‐level epidemiology, but too often abandoned in the publication process by either the researchers, the clinicians, or the media.

It is, however, possible to draw causal inference from non‐intervention observations. The reverse of “association is not causation” is not that “association is never causation”. In fact, observing an association is often the first important first step while causal and non‐causal interpretations are warranted. Causal interpretation of observations mandates careful consideration of methodology, including sources of biases and triangulation of evidence. Astrology, and indeed clinical medicine, would be impossible without pure observational studies. In epidemiology, there are now several advanced study design and analytical methods developed to investigate causality,^3^ but almost inevitably these methods are based on strong assumptions and may require complex statistical analyses. Consequently, clinically relevant studies analyzed with such methods are unfortunately submitted and published less often in clinical journals such as AOGS.

But even descriptive or non‐causal observational studies are profoundly valuable in clinical medicine. Medical doctors interpret the world based on their prior beliefs, update it via patient history and clinical observation, and construct a post‐test probability. For example, if a small head is detected in a routine second‐trimester scan, the post‐test probability of a Zika infection relies heavily on whether the patient has been in an endemic area or not. The Bayesian process of clinical medicine consequently relies on knowledge of the pre‐test prevalence and patterns of disease in the population that can only be obtained through observational studies. Such studies may be considered less “prestigious”, but these findings can still profoundly influence clinical practice.

Importantly, there are clinical and scientific questions for which RCTs would be superior had they not been impossible, unethical, or unfeasible. For example, if one aims to investigate the safety of a new medication in pregnancy that might theoretically cause major malformations, an RCT on such risk would be unethical. Further, even if such an RCT was considered acceptable, the sample size would need to be unrealistically large for specific major malformations (eg the sample size needed to investigate the risk of anencephaly would be over 30 000 under reasonable assumptions). Testing biological complexity would additionally cause an explosion in the needed sample size: if the teratogenic risk of medication is amplified by other medications, which may be the case,^4^ this would call for an unrealistically complex RCT. RCTs are also much less realistic when the research questions involve assessing chronic or long‐term exposure and/or health risk.

That the world, by definition, is more complex than any RCT points to the issue of generalizability. Observational studies may therefore have the upper hand compared to RCTs when it comes to external validity. Again, however, this relies heavily on research methodology and the study conducts. If an observational study is based on flawed design and data with apparent incompleteness, any associations found in the data may have limited value outside the specific context. Further, for the Nordic data often reported on in AOGS, one must focus on the value for, or lack thereof, for populations with, for example, different access to healthcare systems.

Observational studies shine when it comes to hypothesis generation. RCTs are, in essence, hypothesis testing, and even though post hoc analyses of RCTs can lead to new hypotheses, this will be limited compared to the almost endless possibilities of observational data. All these opportunities are, however, also the Achilles' heel of hypothesis‐generating observational studies, and caution is needed when interpreting the possible multitude of associations in such studies. Importantly, null hypothesis significance testing has a different role in hypothesis‐generating studies^5^ particularly in studies on very large data sets where the risk of type I error would be “significant” given no correction for multiple comparisons.

AOGS publishes observational data, including “non‐prestigious” descriptive studies, hypothesis‐generating analyses, or methodologically sound observational studies that interrogate causal relations. In line with the above CEBM statement, however, we need to acknowledge the potential flaws in all studies, observational studies as well as RCTs. The newly updated AOGS statistical guidelines offer some guidance, but ultimately clinicians must ensure that the analytical methods are used and interpreted correctly, and epidemiologists must ensure that the clinical implications are balanced correctly, which may call for interdisciplinary collaborations.

## FUNDING INFORMATION

Dr. Pedersen is supported by a Borregaard Clinical Ascending Investigator Grant from the Novo Nordisk Foundation (NNF18OC0054457). Dr. Liew is supported by the National Institute of Health / National Institute of Child Health and Human Development project award (1R01HD109213‐01A1).
